# A New Notion of Chloroma

**Published:** 1913-12-13

**Authors:** 


### A New Notion of Chloroma.

One of the extremely rare diseases which nearly always provide difficult clinical puzzles when they do occur is that known as chloroma, a condition especially characterised by the development of green tumours on the bones, particularly the skull, and by a progressive anæmia, which is rapidly fatal. In the *Journal of Medical Research* Dr. Burgess advances some new ideas, based on a study of one case and of all the available literature of the condition. He holds that chloroma is by no means a pathological entity as has been commonly supposed. Chloroma tumours, he says, represent a part of the pathological process in one type of acute myelogenous leukæmia; they have not been shown to possess any relation to true lymphatic leukæmia. Myelogenous leukæmia is a blood metastasis, according to this view, of a true tumour whose origin is in the bone marrow, and whose cells invade the blood stream and the bone marrow. In certain cases, however, the cells are relatively undifferentiated, multiply rapidly, and invade other tissues. Sometimes in this way definite nodules of tumour cells are formed, and these masses, if green, are called chloroma.

